# Metabolic changes associated with dark-induced leaf senescence in Arabidopsis *nadk2* mutants

**DOI:** 10.1080/15592324.2023.2215618

**Published:** 2023-06-05

**Authors:** chaomurilege, Atsuko Miyagi, Toshiki Ishikawa, Masatoshi Yamaguchi, Hideki Murayama, Maki Kawai-Yamada

**Affiliations:** a Graduate School of Science and Engineering, Saitama University, Saitama-city, Saitama, Japan; b Faculty of Agriculture, Yamagata University, Tsuruoka-city, Yamagata, Japan

**Keywords:** *Arabidopsis thaliana*, CE-MS, NAD kinase, NAD(P)(H), metabolites

## Abstract

Arabidopsis NADK2 (NAD kinase 2) is a chloroplast-localized enzyme involved in NADP^+^ synthesis, which acts as the final electron acceptor in the photosynthetic electron transfer chain. The NADK2-deficient mutant (nadk2) was used to analyze the effect of NAD(P)(H) unbalance in the dark-induced leaf senescence. During senescence, WT plants and nadk2 mutants showed a similar reduction in chlorophyll content. NAD(P)(H) quantification showed that the amount of total NAD(P)(H) decreased on the day 7 in WT but on the day 3 in nadk2. The phosphorylation ratio (i.e. NADP(H)/NAD(H)) decreased on day 1 in WT. In contrast, the nadk2 showed lower phosphorylation ratio at 0 day and no change throughout the aging process. Metabolome analysis showed that the metabolic profiles of both WT plants and nadk2 mutants subjected to dark-induced senescence adopted similar patterns as the senescence progressed. However, the changes in individual metabolites in the nadk2 mutants were different from those of the WT during dark-induced senescence.

## Introduction

In all organisms, NAD(P)(H) are ubiquitous electron mediators that transfer electrons between oxidized forms (NAD^+^ and NADP^+^) and reduced forms (NADH and NADPH). NADP^+^ and NADPH are typically used in anabolic reactions such as photosynthesis and lipid synthesis, while NAD^+^ and NADH are typically used in catabolic reactions^1^. NAD kinase (NADK) catalyzes the phosphorylation of NAD^+^ to NADP^+^, which modulates a variety of metabolic pathways by altering the phosphorylation ratio (NADP(H)/NAD(H))^2^. Arabidopsis has three different types of the NADK proteins, namely, NADK1 (AT3G21070), NADK2 (AT1G2164), and NADK3 (AT1G78590)^3,4^. NADK1 is located in the cytosol^5^, while NADK3 is located in peroxisomes. Both NADK1 and NADK3 are involved in oxidative stress response^5^. The chloroplast-localized NADK2 plays a critical role in energy transduction in the photosynthesis^6–8^. In previous studies, it has been shown that altering the NADP^+^/NAD^+^ ratio affected the metabolism in both rice and Arabidopsis^9,10^. For example, in rice plants overexpressing NADK2 has increased the NADP^+^/NAD^+^ ratio, and as a result, it has increased the oxidative stress resistance and amino acid accumulation^9,11^. Nonetheless, despite the presence of multiple NADKs that have different subcellular locations and enzymatic properties, their precise physiological role in plants remains largely unknown.

Chloroplast-localized NADK2 is responsible for supplying NADP^+^, the final electron receptor in the photosynthetic electron transfer chain. Under continuous light conditions, T-DNA insertion mutant of NADK2 (*nadk2*) had smaller leaves with pale green color due to a reduction in chlorophyll^6^. Under short-day light conditions, *nadk2* mutants showed more severe growth inhibition and more reactive oxygen species (ROS) accumulation such as hydrogen peroxide compared to the WT plants^12^.

Leaf senescence is accompanied by extensive metabolic transformation, from biosynthesis to degradation. Numerous environmental and developmental factors contribute to leaf senescence, including aging, darkness, hormones, drought, high salinity, and temperature extremes^13^. Dark-induced senescence is used as a model system for studying natural senescence, as it promotes chlorophyll degradation and protein catabolism^14,15^. NAD(P)(H) are involved in both catabolism and anabolism. The phosphorylation ratio of NAD(P)(H), which is regulated by NADK, greatly affects NAD(H)-mediated catabolism and NADP(H)-mediated anabolism. In this study, we focused on leaf senescence, and clarified how *nadk2* mutants undergo dark-induced senescence using metabolome analysis.

## Materials and methods

### Plant materials and growth conditions

In this study, *Arabidopsis thaliana* (ecotype Columbia) was used as the wild type. According to Takahashi et al.^7^, a *nadk2* mutant was obtained from the T-DNA Express Collection at the Salk Institute Genomic Analysis Laboratory (http://signal.salk.edu). In continuous light conditions (70 µmol m^−2^ s^−1^), Arabidopsis seeds were sown directly in Jiffy 7 peat pellets (Jiffy Products International AS, Norway). To induce senescence, two leaves per WT plant and *nadk2* mutant were covered with aluminum foil and kept under dark conditions for 1, 3, and 7 d.

### Measurement of chlorophyll contents

The chlorophyll (a+b) content of the plants was measured by spectrophotometric method. UV absorbances of chlorophyll elution in N, N-dimethylformamide was measured at wavelengths of 647 nm and 664 nm using UV–Vis spectrophotometer (Pharmacia Biotech Ultrospec® 3000 CT, Uppsala, Sweden) and pigment concentrations were calculated according to the method described by Ceusters et al^16^.

### Qrt-PCR analysis

Total RNA was isolated from Arabidopsis leaves using the RNeasy Plant Mini Kit (Qiagen, Venlo, The Netherlands) and DNase I treatment (Qiagen). Reverse transcriptase and a poly dT primer (Thermo Fisher Scientific, Waltham, MA, USA) were used to generate cDNA. Those cDNA samples were analyzed by quantitative real-time PCR (qRT-RCR) using a KAPA SYBR FAST ABI Prism kit (KAPA Biosystems) and an ABI Prism 7500 system (Applied Biosystems). The following primers were used: AT2G29350 (*SAG13*, forward, 5′-CAGCTTGCCCACCCATTGTTA-3′; reverse, 5′-GTCGTACGCACCGCTTCTTTC-3′), AT5G45890 (*SAG12*, forward, 5′-TTGAGCATATAAAAGCGACTG-3′; reverse, 5′-GTGCACTCTCCAGTGAACACA-3′), and AT4G35770 (*SEN1*, forward, 5′-CCACTGCTTTTAACACAACATCA-3′; reverse, 5′-AGCAGTGAGAAGATCAGTTGAGG-3′), while *Actin8* (forward, 5′-TGAGCCAGATCTTCATCGTC-3′, reverse, 5′-TCTCTTGCTCGTAGTCGACA-3′) used as the control.

### Assays of NAD(P)(H) contents

To measure NAD(P)(H), leaves (20–30 mg) were boiled in 200 µL of 0.2 N HCl (to extract of NAD^+^ and NADP^+^) and 0.2 N NaOH (to extract of NADH and NADPH) for 2 min. Samples were homogenized with a handy homogenizer (S-203, AS ONE, Osaka, Japan) and then centrifuged at 15,000 *g* for 10 min at 4°C after sonication (5 sec three times). For NAD^+^ and NADP^+^, 15 µL of 0.2 M NaH_2_PO_4_ (pH 5.6) and 120 µL of 0.2 N NaOH were added. For NADH and NADPH, 15 µL of 0.2 M HEPES/KOH pH 8.0 and 120 µL of 0.2 N HCl were added. NAD(P)(H) contents were measured using a cycling assay as described by Ishikawa et al^17,18^.

### Metabolome analysis

Metabolite extraction was performed according to Miyagi et al. 2020^19^ with minor modification. Approximately 100 mg of frozen leaves were ground by Shake Master Neo Ver. 1.0 (Bio Medical Science, Tokyo, Japan), and 0.15 mL of methanol was added to inactivate enzymes. After adding 0.15 mL of 100 μM piperazine-1,4-bis (2-ethanesulfonic acid) (PIPES) and 100 μM methionine sulfone solution as internal standards, the sample was then centrifuged at 15,000 *g* for 5 min (4°C). The supernatant was then centrifuged (12,000 *g*, 30 min, 4°C) and ultrafiltrated using a 3 kDa cutoff spin column (Merck, Darmstadt, Germany). The filtrate was used for CE-MS analysis.

Metabolites (organic and amino acids) were quantified using CE-triple quadrupole-MS (CE-QqQ-MS) system (CE, 7100; MS, G6420A; Agilent Technologies, Santa Clara, CA, USA) with multi-reaction monitoring (MRM) mode as described by Miyagi et al. 2020^19^. Quantitative accuracy was assessed using Agilent MassHunter Software and known contents of reference substances (approximately 50 primary metabolites).

### Statistical analysis

Multivariate analyses were performed using IBM SPSS v22.0 (IBM, NY, USA). Principal component analysis (PCA) was done using the correlation matrix (cases = each plant; variables = metabolites). The average linkage method (between groups) and the squared Euclidean distance were used in hierarchical clustering analysis (HCA). A heatmap was created using Microsoft Excel 2010 (Microsoft, Redmond, WA, USA). In these analyses, the contents of each metabolite were normalized by the Z-score.

## Results and discussion

### Dark-induced senescence in WT plants and nadk2 mutants

Chloroplast-localized NADK (NADK2) is responsible for providing NADP^+^, a receptor for electrons in the photosynthetic electron transfer chain. The NADK2 knockout mutant (*nadk2*) produces small rosette and pale green leaves as demonstrated in previous studies^6,10,12^. Under short-day light conditions, *nadk2* mutant showed more severe growth inhibition and accumulated more ROS than in the WT plants^12^.

**Figure 1.**
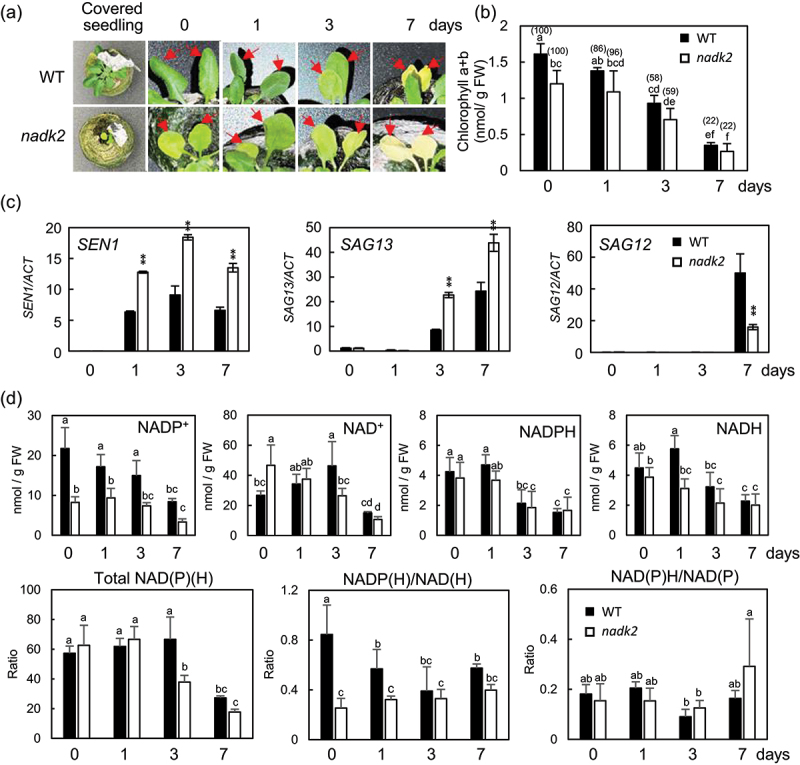
Comparison of dark-induced senescence in the leaves of wild type (WT) plants and nadk2 mutants. (a) Two leaves per plant were covered with aluminum foil and then observed at 1, 3, and 7 d. Red arrows indicate senescence-induced leaves; (b) Chlorophyll contents of senescence-induced leaves were measured at 0, 1, 3, and 7 days (*n* = 3, Tukey’s test, *p* < 0.05). Numbers in parentheses are relative values when day 0 is expressed as 100%. (c) Expression analysis of the senescence-associated genes (SEN1, SAG13 and SAG12) in senescence-induced leaves (0, 1, 3, and 7 d). *n* = 3, **< 0.05. Error bars indicate SD. (d) Comparison of NAD(P)(H) contents and the NAD(P)(H) balance. NAD(P)(H) contents were measured in the senescence-induced leaves at 0, 1, 3, and 7 d in WT plants and nadk2 mutants (*n* = 3, Tukey’s test, *p* < 0.05). Error bars indicate SD. Filled bars indicate WT, and Open bars indicate nadk2 plants.

We also examined the ability of normal dark-induced senescence in the *nadk2* mutant with an abnormal NAD(P)(H) balance. When Arabidopsis leaves were covered with aluminum foil, they turned white in 7 d (Figure 1a). In chlorophyll content analysis, *nadk2* mutants showed lower chlorophyll levels than the WT plants at day 0 as described previously^6,10,12^, and the chlorophyll levels similarly decreased from day 0 to day 7 in both strains (Figure 1b). These data suggested that the WT and *nadk2* leaves similarly undergo dark-induced senescence. However, gene expression associated with senescence (*SEN1*, *SAG13*, and *SAG12*) showed the process of WT and *nadk2* differed (Figure 1c). After senescence-induction, these genes were strongly upregulated as reported previously^20^. The *nadk2* showed twofold increase than those of WT in *SEN1* and *SAG13* expression (day 1–day 7 in *SEN1* and day 3–day 7 in *SAG13*). In contrast, *SAG12* expression in the *nadk2* was 20% of the WT at day 7. The *SAG12* encodes a cysteine protease and *SAG13* encodes a short-chain alcohol dehydrogenase^20^. The *SEN1* gene is encoding unknown functional protein of Arabidopsis and has also been used as a marker to characterize the senescence-associated response^20^. These changes in senescence-related gene expression in the *nadk2* plants support the importance of NAD(P)(H) balance in the process of dark-induced leaf senescence.

A previous study reported that the phosphorylation ratio (i.e., (NADP(H)/NAD(H)) decreased in *nadk2* mutants under normal growth conditions^10^. Thus, the NAD(P)(H) contents were measured in senescence-induced leaves of WT plants and *nadk2* mutants (Figure 1d). The results showed that the amount of NADP^+^ decreased in WT plants at day 7. However, NADP^+^ content in *nadk2* mutants was lower than that in WT plants at day 0 to day 3. A gradual increase in NAD^+^ was observed in WT plants from day 0 to day 3 but decreased by day 7. However, NAD^+^ showed a decreasing trend from day 0 to day 7 in *nadk2* mutant. NADPH contents in both strains were not significantly different at any time point. NADH content in the *nadk2* mutant was considerably lower than that of the WT plants on day 1. As the senescence progressed, total NAD(P)(H) contents decreased on the day 7 in WT but decreased on the day 3 in *nadk2*. The phosphorylation ratio (i.e., NADP(H)/NAD(H)) decreased in WT on day 1. In contrast, the *nadk2* showed lower phosphorylation ratio at 0 day, and it was not changed at all the time points examined. There were no clear changes in the redox ratio (i.e., (NAD(P)H/NAD(P)) of WT plants and *nadk2* mutants (Figure 1d).

NADK2 regulates the phosphorylation ratio of NAD(P)(H), which in turn maintains NAD(H) levels during catabolism and NADP(H) during anabolism. The defect in NADK2 caused difference in the decrease in the NAD(P)(H) contents and phosphorylation ratio of WT plants and *nadk2* mutants but not the redox ratio of NAD(P)(H) balance during dark-induced senescence. We therefore examined what metabolite changes are seen during dark-induced senescence in the *nadk2* mutant.

### Nadk2 mutants showed altered metabolite profiles during dark-induced senescence

**Figure 2.**
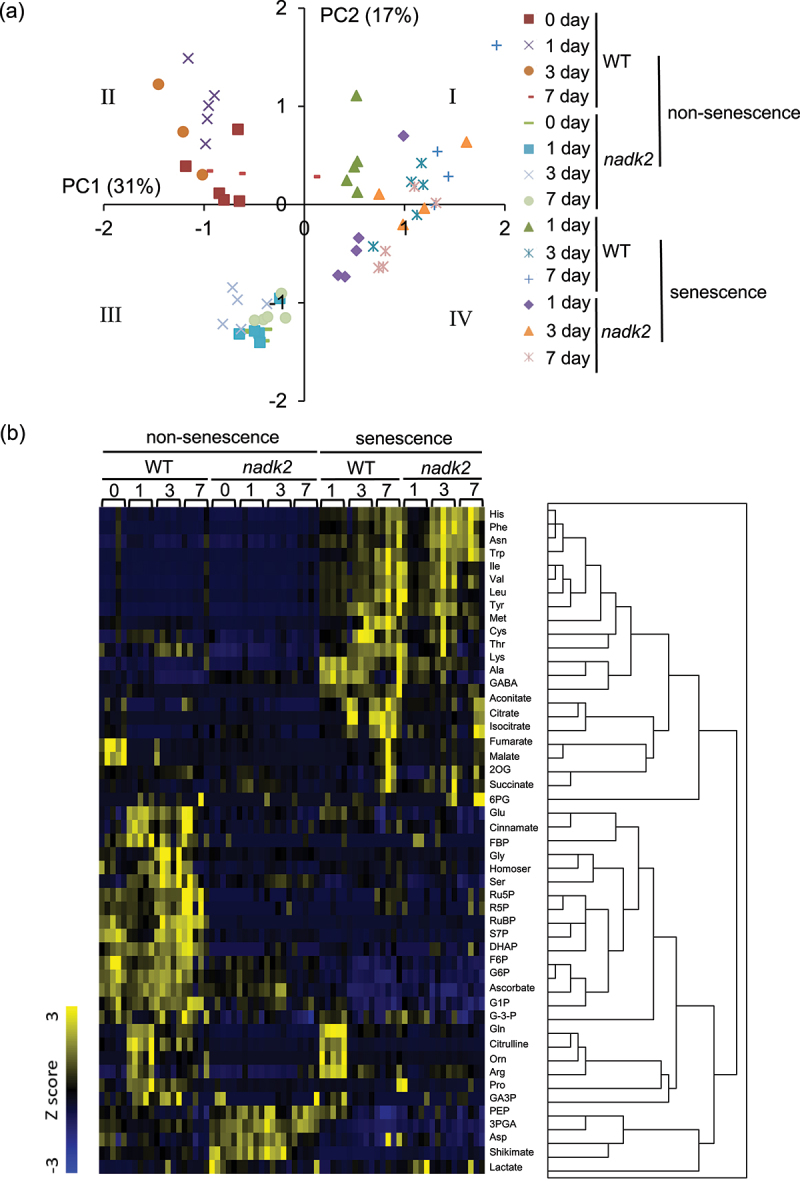
Metabolome analysis in non-senescent leaves and dark-induced senescent leaves. (a) PCA of metabolic data obtained from wild-type (WT) plants and nadk2 mutants; (b) a dendrogram obtained by HCA (right) and a heat map (left) of metabolites in WT plants and nadk2 mutants. HCA was performed using the Z score. Each column of the heat map corresponds to an individual experiment; *n* = 5.

**Figure 3.**
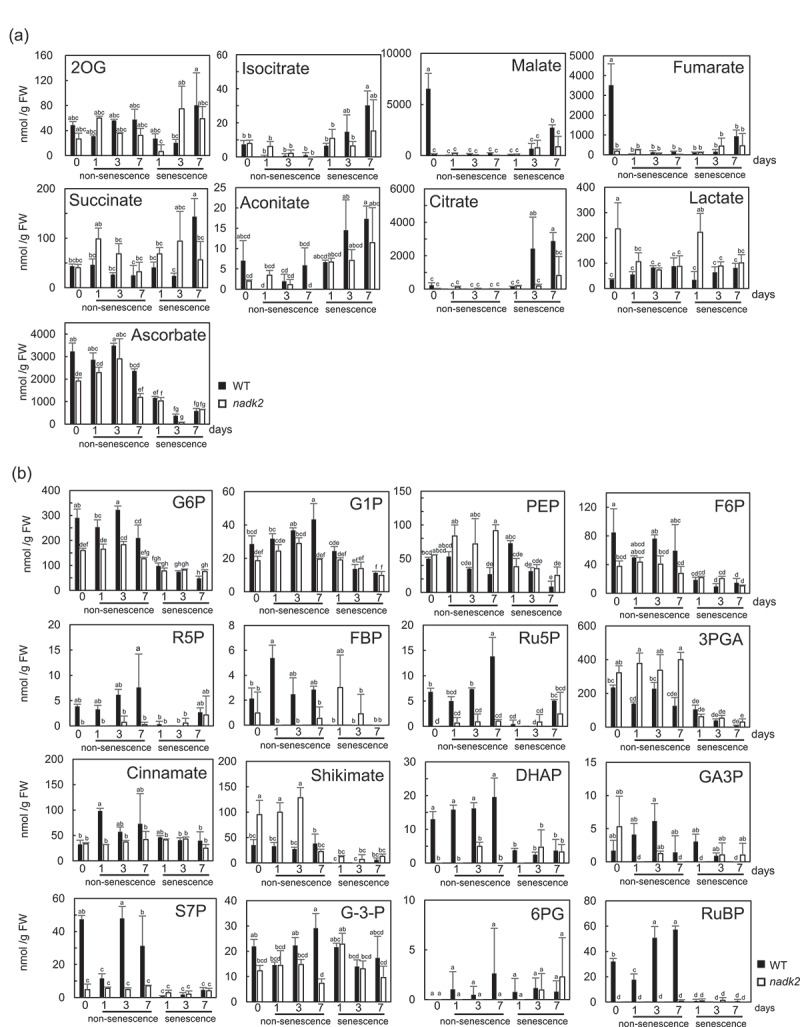
Quantitative comparison of organic acids (a) and sugar phosphates (b) in non-senescent and dark-induced senescent leaves of wild-type (WT) plants and nadk2 mutants (*n* = 3, Tukey’s test, *p* < 0.05). Error bars indicate SD. Filled bars indicate WT, and Open bars indicate nadk2 plants.

**Figure 4.**
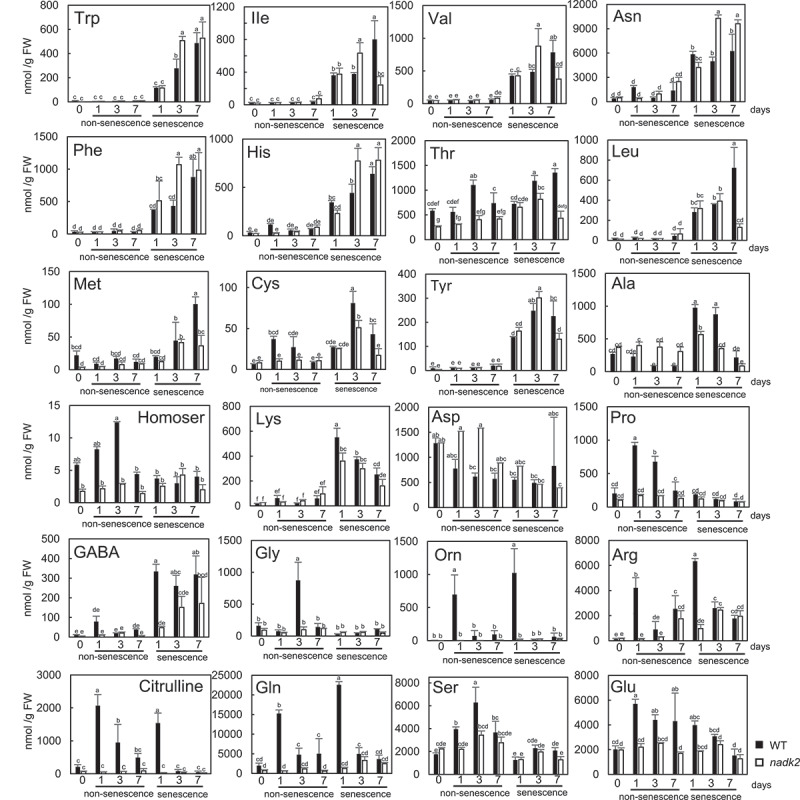
Quantitative comparison of amino acids in non-senescent and dark-induced senescent leaves of wild-type (WT) plants and nadk2 mutants (*n* = 3, Tukey’s test, *p* < 0.05). Error bars indicate SD. Filled bars indicate WT, and Open bars indicate nadk2 plants.

**Figure 5.**
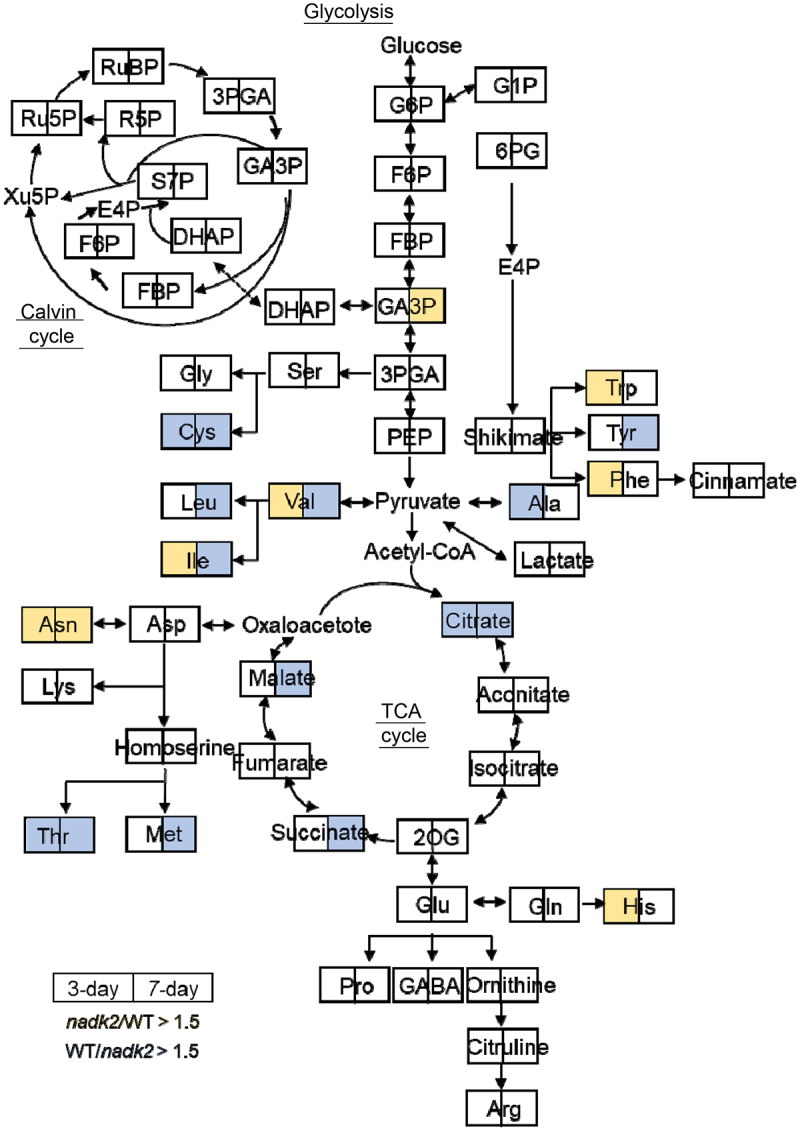
Metabolic changes in nadk2 leaves during dark-induced senescence. Metabolites on day 3 (left boxes) and day 7 (right boxes) after senescence induction were shown. Colors indicates increased (orange) or decreased (blue) in nadk2 compared with WT plants. Only metabolites with statistically significant differences in Figure 3 and Figure 4 are shown.

The metabolomic alterations resulted by dark-induced senescence were analyzed in WT and *nadk2* mutant plants using CE-MS. Forty-nine metabolites involved in fundamental metabolic processes such as glycolysis, TCA cycle, Calvin cycle and the pentose phosphate pathway were detected in non-senescent and dark-induced senescent leaves. PCA and HCA were used to visualize the differences of metabolic alterations between WT and *nadk2* mutants (Figure 2a, b). Two principal components, PC1 (31%) and PC2 (17%), together accounted for 48% of variance in the metabolite dataset (Figure 2a). The results of the PCA showed that the metabolic patterns of the WT plants and *nadk2* mutants were quite different in the non-senescent leaves at day 0, as reported previously^10,21^. In the non-senescent leaves, different metabolic profiles were observed at day 1, 3, and 7. As opposed to that, in leaves subjected to dark-induced senescence, the metabolic profiles of the WT plants and *nadk2* mutants moved toward the first quadrant (Figure 2a). The WT plants and *nadk2* mutants were highly different in their initial metabolic states, but after the induction of dark-induced senescence, the metabolic profiles of both the WT plants and *nadk2* mutants were almost similar except several individual metabolites (Figures 3–5). Lactate was significantly increased in the non-senescent leaves of *nadk2* mutants at day 0 and in dark-induced senescent leaves of *nadk2* mutants at day 1. Malate level at day 7 and citrate level at both day 3 and day 7 in senescent leaves of *nadk2* mutants were lower than that of in WT. The succinate levels in senescent leaves were also lower in *nadk2* mutants at day 7 (Figures 3a, and 5).

In day 0, sugar phosphate metabolite groups, such as glucose-6-phosphate (G6P), fructose 6-phosphate (F6P), ribulose-5-phosphate (Ru5P), dihydroxyacetone phosphate (DHAP), sedoheptulose-7-phosphate (S7P), and ribulose-1,5-bisphosphate (RuBP) showed higher amounts in WT plants than in *nadk2* mutants (Figure 3b). These metabolites showed decrease in senescent leaves at day 1 in WT, and this decrease appears to be associated with photosynthetic activity. According to a previous study, a decrease in photosynthetic activity in rice leaves has been attributed to leaf senescence^22^.

Numerous amino acids showed an increase in dark-induced senescent leaves (Figures 4 and 5). In particular, a marked increase was observed in tryptophan (Trp), isoleucine (Ile), valine (Val), asparagine (Asn), phenylalanine (Phe) and histidine (His) in the *nadk2* mutants compared to the WT plants at day 3. However, cysteine (Cys), threonine (Thr), and alanine (Ala) were lower in *nadk2* mutants than WT at day 3. Focusing on dark-induced senescence day 7, asparagine (Asn) levels were increased in the *nadk2* mutants, while methionine (Met), Cys, Thr, leucine (Leu), tyrosine (Tyr), Ile and Val levels were lower in the *nadk2* mutants. It has been reported that the proteins in senescent leaves are degraded into their constituent amino acids, which are then transported elsewhere in the plant^23^. As shown in Figure 5, at the later stage of senescence (day 3 and day 7), most sugar phosphates involved in Calvin cycle showed no difference between WT and *nadk2* plants. Conversely, amino acids and organic acids showed large differences, suggesting that metabolism related to protein degradation and respiration has been affected in *nadk2* mutants.

According to Law et al.^24^, gene encoding for proteins involved in primary energy production (respiration, fermentation, and oxidation), amino acid, lipid, or nucleotide catabolism, sulfur metabolism, and shikimate metabolism are all overexpressed during senescence in darkened leaves. Thus, senescence is not considered to be a passive death step, but an important metabolic process that requires active metabolism. Consequently, the reduced phosphorylation ratio in the NAD(P)(H) balance may affect a wide range of metabolic pathways.

There was a trend toward reduced amounts of sugar phosphates in senescent leaves compared to non-senescent leaves, whereas the intermediates of the TCA cycle showed a rather complex pattern. This complex pattern may reflect the role of these intermediates as alternative respiratory substrates during the progression of senescence^25,26^. Previous studies have shown that the metabolism of C and N is markedly reduced in *nadk2* mutants^9,10^. Differences in metabolic profiles in non-senescent leaves may be responsible for the differences in the intermediate processes and final products in dark-induced senescent leaves.

## Conclusion

In this study, we investigated changes in NAD(P)(H) balance and metabolites in *nadk2* mutants during dark-induced leaf senescence. The results showed that individual metabolites of Arabidopsis WT plants and *nadk2* mutants responded differently to the induction of senescence. In particular, *nadk2* mutants showed large differences in the amino acid contents produced from protein-degradation during senescence. Further studies will be important to clarify the effects of differences in the NAD(P)(H) balance on the degradation and translocation of substances in dark-induced senescence leaves in Arabidopsis WT plants and *nadk2* mutants.

## Abbreviations

CE-MS: capillary electrophoresis-mass spectrometry
DHAP: dihydroxyacetone phosphate
F6P: fructose 6-phosphate
G1P: glucose-1-phosphate
G-3-P: glycerol 3-phosphate
G6P: glucose-6-phosphate
HCA: hierarchical clustering analysis
NADP^+^: nicotinamide adenine dinucleotide phosphate
NAD^+^: nicotinamide adenine dinucleotide
PCA: Principal component analysis
PEP: phosphoenolpyruvate
3PGA: 3-phosphoglycerate
R5P: ribose-5-phosphate
RuBP: ribulose-1,5-bisphosphate
Ru5P: ribulose-5-phosphate
S7P: sedoheptulose-7-phosphate
TCA: tricarboxylic acid
WT: wild type

## References

[1] Hashida SN, Takahashi H, Uchimiya H. The role of NAD biosynthesis in plant development and stress responses. Ann Bot. 2009;103(6):819–9. doi:10.1093/aob/mcp019. 19201765 PMC2707885

[2] Grose JH, Joss L, Velick SF, Roth JR. Evidence that feedback inhibition of NAD kinase controls responses to oxidative stress. Proc Natl Acad Sci USA. 2006;103(20):7601–7606. doi:10.1073/pnas.0602494103. 16682646 PMC1472491

[3] Berrin J-G, Pierrugues O, Brutesco C, Alonso B, Montillet J-L, Roby D, Kazmaier M. Stress induces the expression of AtNADK-1, a gene encoding a NAD (H) kinase in *Arabidopsis thaliana*. Mol Genet Genom. 2005;273(1):10–19. doi:10.1007/s00438-005-1113-1. 15711971

[4] Turner WL, Waller JC, Snedden WA. Identification, molecular cloning and functional characterization of a novel NADH kinase from *Arabidopsis thaliana* (thale cress). Biochem J. 2005;385(1):217–223. doi:10.1042/BJ20040292. 15347288 PMC1134690

[5] Chai M-F, Wei P-C, Chen Q-J, An R, Chen J, Yang S, Wang X-C. NADK3, a novel cytoplasmic source of NADPH, is required under conditions of oxidative stress and modulates abscisic acid responses in Arabidopsis. Plant J. 2006;47(5):665–674. 10.1111/j.1365-313X.200602816.x. 16856986

[6] Chai M-F, Chen Q-J, An R, Chen Y-M, Chen J, Wang X-C. NADK2, an Arabidopsis chloroplastic NAD kinase, plays a vital role in both chlorophyll synthesis and chloroplast protection. Plant Mol Biol. 2005;59(4):553–564. doi:10.1007/s11103-005-6802-y. 16244906

[7] Takahashi H, Watanabe A, Tanaka A, Hashida S-N, Kawai-Yamada M, Sonoike K, Uchimiya H. Chloroplast NAD kinase is essential for energy transduction through the xanthophyll cycle in photosynthesis. Plant Cell Physiol. 2006;47(12):1678–1682. doi:10.1093/pcp/pcl029. 17082216

[8] Turner WL, Waller JC, Vanderbeld B, Snedden WA. Cloning and characterization of two NAD kinases from Arabidopsis. Identification of a calmodulin binding isoform. Plant Physiol. 2004;135(3):1243–1255. doi:10.1104/pp.104.040428. 15247403 PMC519044

[9] Takahara K, Kasajima I, Takahashi H, Hashida SN, Itami T, Onodera H, Toki S, Yanagisawa S, Kawai-Yamada M, Uchimiya H. Metabolome and photochemical analysis of rice plants overexpressing Arabidopsis NAD kinase gene. Plant Physiol. 2010;152(4):1863–1873. doi:10.1104/pp.110.153098. 20154096 PMC2850022

[10] Takahashi H, Takahara K, Hashida SN, Hirabayashi T, Fujimori T, Kawai-Yamada M, Yamaya T, Yanagisawa S, Uchimiya H. Pleiotropic modulation of carbon and nitrogen metabolism in Arabidopsis plants overexpressing the NAD kinase2 gene. Plant Physiol. 2009;151(1):100–113. doi:10.1104/pp.109.140665. 19587098 PMC2735975

[11] Onda Y, Miyagi A, Takahara K, Uchimiya H, Kawai‐yamada M, Mendel R. Effects of NAD kinase 2 overexpression on primary metabolite profiles in rice leaves under elevated carbon dioxide. Plant Biol. 2014;16(4):819–824. doi:10.1111/plb.12131. 24397549

[12] Chaomurilege ZY, Miyagi A, Hashida SN, Ishikawa T, Yamaguchi M, Kawai‐yamada M, Kawai-Yamada M. Loss of chloroplast‐localized NAD kinase causes ROS stress in *Arabidopsis thaliana*. J Plant Res. 2023;136(1):97–106. doi:10.1007/s10265-022-01420-w. 36367584

[13] Lim PO, Kim HJ, Nam HG. Leaf senescence. Annu Rev Plant Biol. 2007;58(1):115–136. doi:10.1146/annurev.arplant.57.032905.105316. 17177638

[14] Weaver LM, Gan S, Quirino B, Amasino RM. A comparison of the expression patterns of several senescence-associated genes in response to stress and hormone treatment. Plant Mol Biol. 1998;37(3):455–469. doi:10.1023/A:1005934428906. 9617813

[15] Guo Y, Gan S. Leaf senescence: signals, execution, and regulation. Curr Top Dev Biol. 2005;71:83–112. doi:10.1016/S0070-2153(05)71003-6. 16344103

[16] Ceusters N, Valcke R, Frans M, Claes JE, Van den Ende W, Ceusters J. Performance index and PSII connectivity under drought and contrasting light regimes in the CAM orchid Phalaenopsis. Front Plant Sci. 2019;10:1012. doi:10.3389/fpls.2019.01012. 31447875 PMC6691161

[17] Ishikawa Y, Miyagi A, Haishima Y, Ishikawa T, Nagano M, Yamaguchi M, Hihara Y, Kawai-Yamada M. Metabolomic analysis of NAD kinase-deficient mutants of the cyanobacterium *Synechocystis* sp. PCC 6803. J Plant Physiol. 2016;205:105–112. doi:10.1016/j.jplph.2016.09.002. 27657983

[18] Ishikawa Y, Kawai-Yamada M, Hashida SN. Measurement of chloroplastic NAD kinase activity and whole tissue NAD kinase assay. Bio-protocol. 2020;10(1):e3480. doi:10.21769/BioProtoc.3480. 33654713 PMC7842763

[19] Miyagi A, Saimaru T, Harigai N, Oono Y, Hase Y, Kawai‑yamada M. Metabolome analysis of rice leaves to obtain low-oxalate strain from ion beam-mutagenised population. Metabolomics. 2020;16(9):94. doi:10.1007/s11306-020-01713-y. 32894362

[20] James M, Poret M, Masckaux-Daubresse C, Marmagne A, Coquet L, Jouenne T, Chan P, Trouverie J, Etienne P. SAG12, a major cysteine protease involved in nitrogen allocation during senescence for seed production in Arabidopsis thaliana. Plant & Cell Physiol. 2018;59(10):2052–2063. doi:10.1093/pcp/pcy125. 29982633

[21] Kawai-Yamada M, Miyagi A, Sato Y, Hosoi Y, Hashida S-N, Ishikawa T, Yamaguchi M. Altered metabolism of chloroplastic NAD kinase- overexpressing Arabidopsis in response to magnesium sulfate supplementation. Plant Signal Behav. 2021;16(1):1844509. doi:10.1080/15592324.2020.1844509. 33210985 PMC7781788

[22] Kura-Hotta M, Satoh K, Katoh S. Relationship between photosynthesis and chlorophyll content during leaf senescence of rice seedlings. Plant Cell Physiol. 1987;28:1321–1329. doi:10.1093/oxfordjournals.pcp.a077421.

[23] Mae T, Ohira K. The remobilization of nitrogen related to leaf growth and senescence in rice plants (*Oryza sativa* L.). Plant Cell Physiol. 1981;22:1067–1074. doi:10.1093/oxfordjournals.pcp.a076248.

[24] Law SR, Chrobok D, Juvany M, Delhomme N, Lindén P, Brouwer B, Ahad A, Moritz T, Jansson S, Gardeström P, et al. Darkened leaves use different metabolic strategies for senescence and survival. Plant Physiol. 2018;177(1):132–150. doi:10.1104/pp.18.00062. 29523713 PMC5933110

[25] Araújo WL, Ishizaki K, Nunes-Nesi A, Larson TR, Tohge T, Krahnert I, Witt S, Obata T, Schauer N, Graham IA, et al. Identification of the 2-hydroxyglutarate and isovaleryl-CoA dehydrogenases as alternative electron donors linking lysine catabolism to the electron transport chain of Arabidopsis mitochondria. Plant Cell. 2010;22(5):1549–1563. doi:10.1105/tpc.110.075630. 20501910 PMC2899879

[26] Araújo WL, Tohge T, Ishizaki K, Leaver CJ, Fernie AR. Protein degradation – an alternative respiratory substrate for stressed plants. Trends Plant Sci. 2011;16(9):489–498. doi:10.1016/j.tplants.2011.05.008. 21684795

